# Light-driven single-cell rotational adhesion frequency assay

**DOI:** 10.1186/s43593-022-00020-4

**Published:** 2022-08-08

**Authors:** Yaoran Liu, Hongru Ding, Jingang Li, Xin Lou, Mingcheng Yang, Yuebing Zheng

**Affiliations:** 1 Department of Electrical and Computer Engineering, The University of Texas at Austin, Austin, TX 78712 USA; 2 Walker Department of Mechanical Engineering, The University of Texas at Austin, Austin, TX 78712 USA; 3 Materials Science & Engineering Program and Texas Materials Institute, The University of Texas at Austin, Austin, TX 78712 USA; 4 School of Physical Sciences, University of Chinese Academy of Sciences, Beijing, 100049 China; 5 Beijing National Laboratory for Condensed Matter Physics and Laboratory of Soft Matter Physics, Institute of Physics, Chinese Academy of Sciences, Beijing, 100190 China; 6 Songshan Lake Materials Laboratory, Dongguan, 523808 Guangdong China; 7 Department of Biomedical Engineering, The University of Texas at Austin, Austin, TX 78712 USA

## Abstract

The interaction between cell surface receptors and extracellular ligands is highly related to many physiological processes in living systems. Many techniques have been developed to measure the ligand-receptor binding kinetics at the single-cell level. However, few techniques can measure the physiologically relevant shear binding affinity over a single cell in the clinical environment. Here, we develop a new optical technique, termed single-cell rotational adhesion frequency assay (scRAFA), that mimics in vivo cell adhesion to achieve label-free determination of both homogeneous and heterogeneous binding kinetics of targeted cells at the subcellular level. Moreover, the scRAFA is also applicable to analyze the binding affinities on a single cell in native human biofluids. With its superior performance and general applicability, scRAFA is expected to find applications in study of the spatial organization of cell surface receptors and diagnosis of infectious diseases.

**Supplementary Information:**

The online version contains supplementary material available at 10.1186/s43593-022-00020-4.

## Introduction

**Fig. 1:**
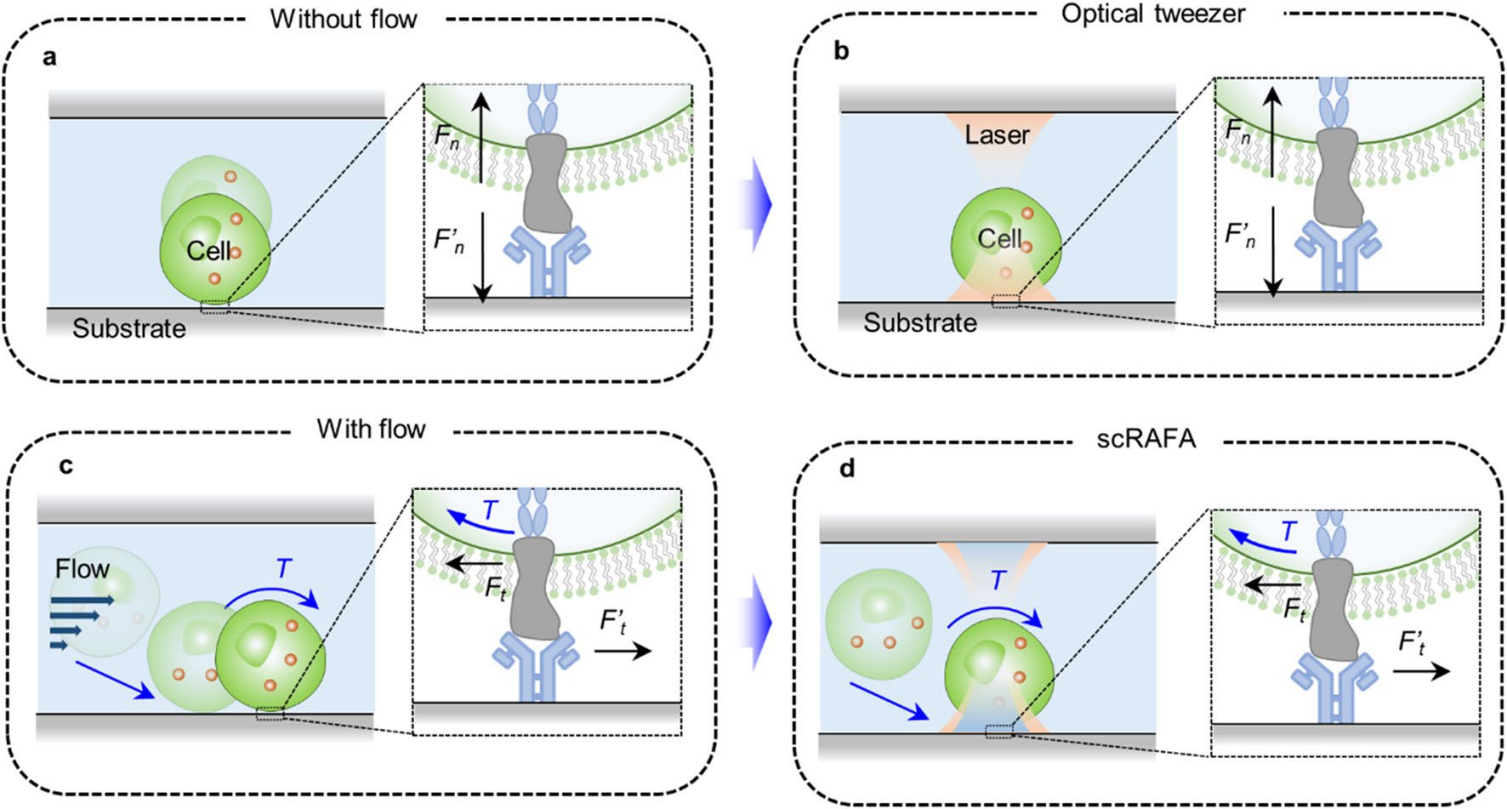
Schematic illustration of the concept of scRAFA and its advantage over the existing assays in measuring shear adhesion kinetics. **a** Without a fluid flow, a cell adheres to a functionalized substrate with a binding force along the normal direction of the substrate. To detach the cell from the substrate, a tensile force is applied to break up the bond between the cell receptor and substrate ligand and. **b** Conventional single-cell adhesion assays (i.e., optical tweezers, magnetic tweezers, adhesion frequency assays, and AFM), which apply different forces to rupture the adhesive contact, measure the tensile force (*F*_*n*_*,* *F’*_*n*_) along the normal direction of the cell-substrate interface. **c** With a biofluid (i.e., blood, urine, etc.) flow, the adhesion of a cell experiences multiple stages. The cell will first attach to the endothelium cells (as a substrate) at an inclined angle. Some cells can instantaneously adhere to the substrate during this stage while others will roll along the endothelium cells through transient cell–cell interactions. During the cell rolling, the interacting molecules (i.e., receptors on a cell and ligands on a substrate) can experience the shear forces (*F*_*t*_*,* *F’*_*t*_). An arrow with “*T*” indicates the torque on the cell generated by the fluidic flow. **d** In scRAFA, we first trap the cell closer to the functionalized substrate from the liquid medium, which mimics the cell pre-attachment as shown in the first stage of **c**. For those cells that do not instantaneously adhere to the substrate, we achieve the measurement of their shear adhesion kinetics through analyzing the light-driven out-of-plane rotation of the cells near the substrate

The ligand-receptor binding is highly relevant to many biological processes such as leukocytes-mediated immunity and infectious disease [1–5]. For example, the leukocyte can enter the injured tissue by binding to the P selectin on the endothelial cells [6]. COVID-19 is caused through the binding between viral spikes and angiotensin-converting enzyme 2 (ACE2) on the host cells [7]. Several types of single-cell adhesion assays have been developed to study the binding or adhesion kinetics. Adhesion frequency assays, which employ two micropipettes to precisely control the contact between one sensing cell and one target cell during the pulling-pushing process, can measure the adhesion kinetics by repeatedly rupturing the adhesive contact of the cells [7, 8]. Other techniques based on atomic force microscopy (AFM), optical tweezers or magnetic tweezers can measure the adhesion forces between cells down to single-receptor resolution [9, 10]. They can even achieve three-dimensional mapping of the adhesion force distribution on single cells. However, the difference in the measured affinities of P selectin and ACE2 using different methods can be as large as four orders [11] and one order of magnitude [12, 13], respectively. The difference arises from the factor that the measured samples had variations in the length of interacting molecules, the elasticity of linker molecules, or the scheme of molecular immobilization on surfaces, which affected the bond dissociation kinetics. Meanwhile, the existing methods based on rupturing the adhesive contact at the microscale are mainly measuring the tensile force along the normal direction of the cell interface (Fig. 1a, b), which is far from the actual in vivo cell adhesion condition.

In vivo cell adhesion under the physiological flow is more complex than what the existing methods can measure. At an initial stage, a cell will pre-attach to the endothelium cells in an inclined direction. After the initial attachment, the cell will experience fluid-flow-induced shear force, enabling the receptor and ligand to slide against each other [14]. The sliding promotes the formation of new interactions after the pre-existing attachment is ruptured [15]. In this case, the force direction between ligand and receptor is tangential to the cell or parallel to the substrate, leading to different cell adhesion behavior (Fig. 1c). Preliminary studies have shown that integrins require lateral rather than normal force to mediate cell–cell interactions [16]. Many T-cell receptors (TCR) can generate collectively tangential force at the contact interface [15]. So far, few techniques have been developed to analyze the lateral force along the tangential direction. Microfluidic chambers have been used to study this condition with pump-controlled shear flow [17, 18]. However, the flow chamber assay cannot precisely control the cell-substrate distance for biologically relevant interactions or target a specific cell due to the random distribution of many cells in the flow. It is limited to analyzing the adhesion kinetics of a fraction of cells that randomly undergo rolling processes under the microfluidic flow. In addition, one cannot control the adhesion measurement time with the flow chamber assay, which is usually equivalent to the period from cell contacting the chamber wall to cell forming the initial bonding with the wall, limiting the precision of the cell adhesion analysis.

To overcome the limits of existing assays for cell adhesion measurements, we develop a light-driven single-cell rotational adhesion frequency assay (scRAFA), which enables label-free and sub-cellular-resolution quantification of adhesion of almost any targeted individual cells in clinical solutions (Fig. 1d). Rather than measuring the adhesion kinetics by rupturing the adhesive contact in the normal direction, our scRAFA measures the adhesion kinetics of cells that undergo light-driven out-of-plane rotation near the functionalized substrate. Through a seamless fusion of optical rotation and trapping on a single platform, we can target almost any specific cells, continuously monitor the complete cell adhesion process from initiating the bonding with the substrate to forming the permanent attachment, and precisely control the interaction distance between the substrate ligands and cell receptors for measuring the lateral adhesion kinetics, which cannot be achieved by the conventional methods. As such, scRAFA enables in situ high-precision measurement of the shear adhesion kinetics on targeted cells in complex clinical samples.

## Results

**Fig. 2:**
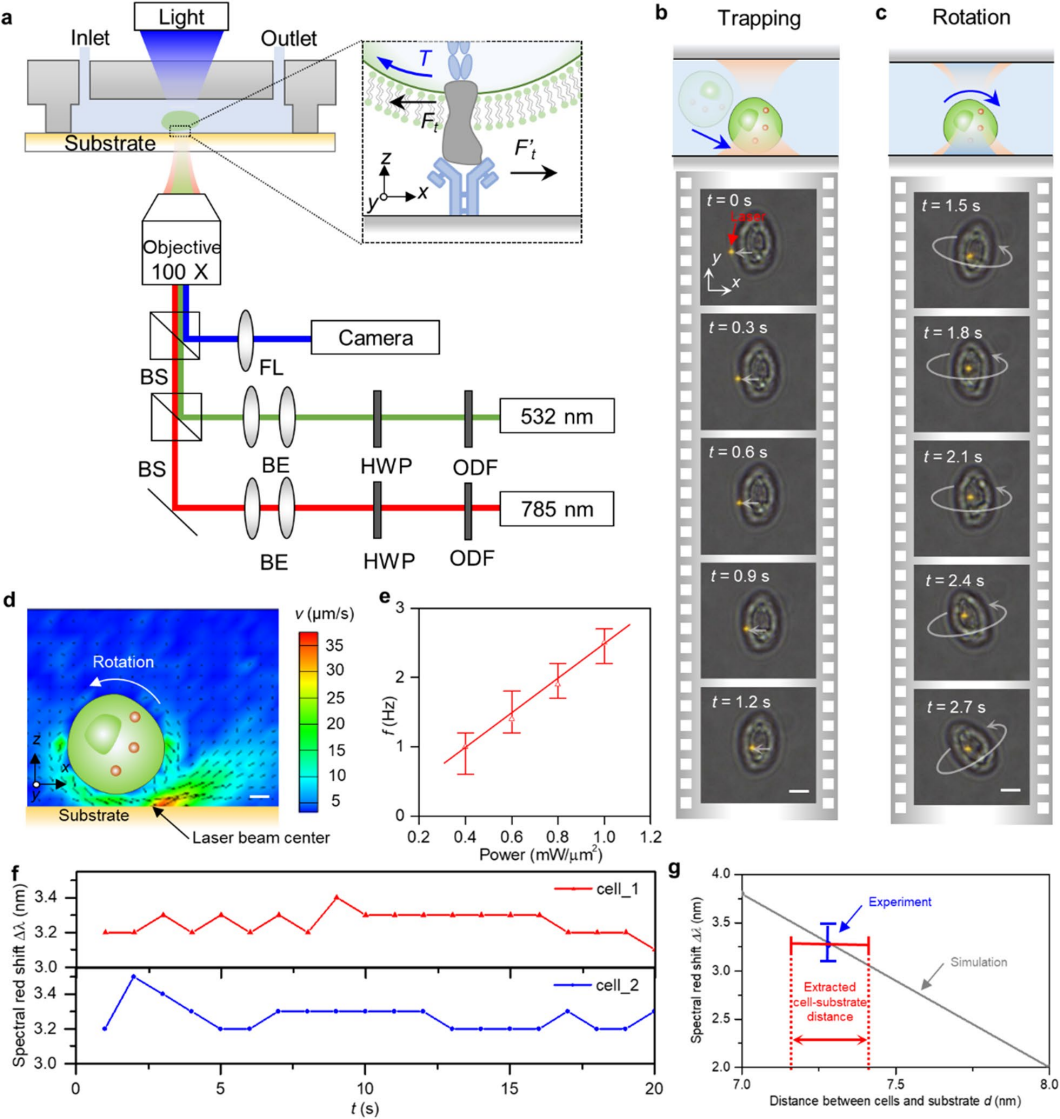
Experimental setup and working principle of scRAFA. **a** Experimental setup of scRAFA. Two laser beams with wavelengths of 785 nm and 532 nm are focused at the same position on the substrate to trap and rotate a single cell. The inset illustrates that the receptors of the rotating cell interact with ligand molecules immobilized on the substrate. The motion of the cell is monitored by in situ optical imaging through a digital camera. BS: beam splitter. BE: beam expander. HWP: half-wave plate. ODF: optical density filter. FL: focused lens. **b**, **c** A sequence of optical images of the trapping and rotation of a single cell. Scale bars: 5 μm. **d** Cross-sectional view of the flow velocity near the light-absorbing substrate where the optically trapped cell undergoes out-of-plane rotation. Scale bar: 1 μm. **e** The rotational frequency of the trapped cell under different optical power of 532 nm laser. The optical power of 785 nm laser is 1 mW/μm^2^ in all studies. **f** Time-dependent experimental spectral red shift collected from a rotating cell. **g** Simulated spectral red shift (*Δλ*) with different distances between the cell and the substrate (*h*) (gray line) along with the experimental spectral red shifts (blue line) and the extracted cell-substrate distance (red line). The red double arrow corresponds to the extracted cell-substrate distance

Our scRAFA exploits a microfluidic platform integrated with versatile optothermal manipulation and optical imaging to trap and rotate single cells while monitoring the sequential cell rotation and cell-substrate adhesion [19, 20] (Fig. 2a). As a demonstration, Figs. 2b, c show the successive images of light-driven trapping and rotation of a single *S.* *cerevisiae* above a substrate (Additional file 2: Movie S1). We achieve high-efficient trapping and rotation of the targeted cell using two working laser beams with wavelengths of 785 nm and 532 nm. The substrate is designed to strongly absorb the 532 nm laser beam while being mostly transparent to the 785 nm laser beam. Specifically, a focused 785 nm laser beam is applied to trap the cell with optical force [21–23]. To achieve the stable rotation of the cell while being trapped, we further apply a focused 532 nm laser beam to heat the light-absorbing substrate near the trapped cell to generate a temperature gradient field (Additional file 1: Fig. S1), where thermophoretic force repels the cell from the laser-heated hot region while thermo-osmotic force attracts the cell to the hot region [24–26]. Thermo-osmosis is a surface-driven effect where a temperature gradient along the substrate induces a thermo-osmotic flow that is parallel to the substrate. With the increased laser power, the temperature gradient along the substrate increases and hence results in a stronger thermo-osmotic flow velocity. A balance among the optical force, thermophoretic force, and thermo-osmotic force leads to a stable optothermal trapping of the cell at the side of the heating laser beam (Additional file 1: Fig. S2). Without the optical force from the 785 nm laser beam, the 532 nm-laser-induced thermophoretic force and thermo-osmotic force cannot achieve stable trapping of the cells (Additional file 1: Fig. S2). Moreover, we manage an unbalanced thermo-osmotic flow along the cell surface to exert a torque on the trapped cell and to optothermally drive the out-of-plane rotation of the cell (Fig. 2d) [27]. The rotational speed is proportional to the heating laser power (Fig. 2e). To quantify the force on the cell, we extract the rotational speed of the cell from the video and calculate the force through Stokes’ law. Specifically, when the optical power of 532 nm and 785 nm lasers is 0.2 mW/μm^2^ and 1 mW/μm^2^, the corresponding rotational frequency is ~ 1 Hz and the calculated shear force is ~ 4 pN for 5 μm cell [28]. Similar to our previously theoretical analysis [27], the rotation is anticlockwise for a cell trapped at the left sides of the laser beam. To further verify the mechanism for optothermal manipulation, we experimentally track the central position of a cell being trapped and rotated relative to the laser beam center. The temporal trajectory distribution of the cell center shows that the stable cell-trapping position is away from the laser center (Additional file 1: Fig. S3), which matches well with our force analysis (Additional file 1: Fig. S2). In contrast to conventional optical tweezers where the cell is trapped at the focused laser beam center, the stable trapping position of the cell in our assay is away from the laser beam center. The distance between the heating center and the trapped cell center is over 2 μm due to the repelling thermophoretic force. The actual temperature increase on the cell membrane is below 10 degrees when the laser power is 0.2 mW/μm^2^, which causes much less thermal damage to the cell (Additional file 1: Fig. S4).

Precise control of the interacting distance between cell and substrate is pivotal for measuring the adhesion strength in our scRAFA due to the strong dependence of the adhesion strength on the cell-substrate distance, which can be precisely controlled by moving the trapped cell up and down through optical tweezer. We conduct in situ optical transmission measurements to extract the cell-substrate distance during the cell rotation (Additional file 1: Note 1) [29]. As an example, we collected the time-dependent transmission spectral shift for cell 1 and cell 2 during cell rotation (Fig. 2f). The closer distance between the cells and substrate, the larger the red shift observed in the transmission dip. The distance between the cell and the substrate can be extracted once there are Δλ matches between the simulated and experimental results (Fig. 2g). By sweeping the distance between cells and substrates *d* in the simulation, we show that the spectral shift has different values. Specifically, when *d* =  ~ 7.4 nm, the simulated spectral red shift matches with the experimental data. Therefore, the distance between the cell and substrate is ~ 7.4 nm ± 0.1 nm, indicating that our technique can precisely control the interacting distance between cell and substrate. Different from cells with rough surfaces like neutrophils, the cell in the current study is topologically spherical and homogeneous. This is consistent with the previous AFM measurement, which revealed the surface roughness of *S.* *cerevisiae* as a root mean square of ~ 0.3 nm [30]. Therefore, our rotational adhesion measurements on the spherical cells can preclude the inaccuracy caused by the anisotropic cell shape.

**Fig. 3:**
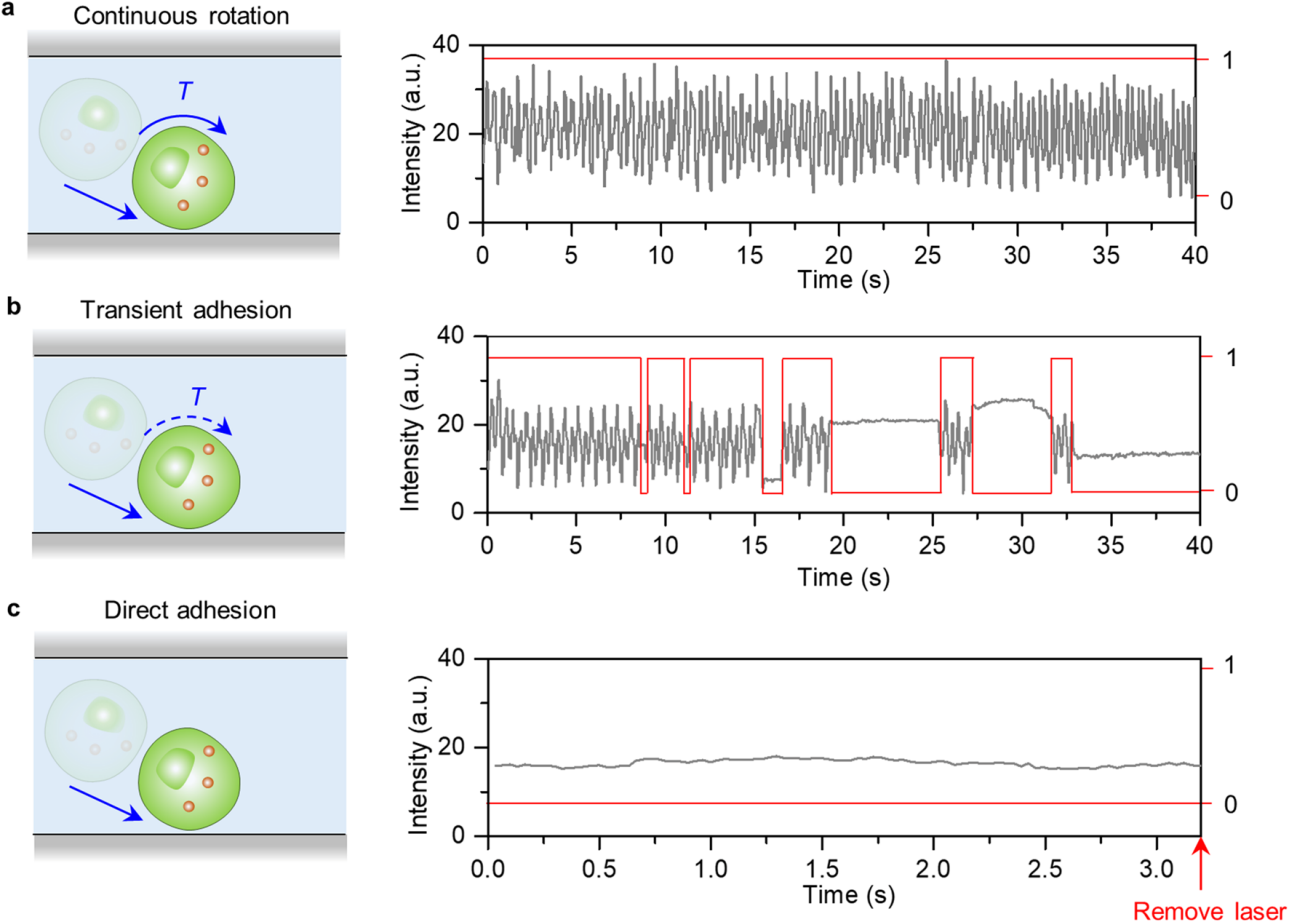
Three cell behaviors observed during the optical trapping. Schematics (left panel) and time-dependent image intensity (right panel) collected from a trapped cell undergoing **a** continuous rotation, **b** transient adhesion, and **c** direct adhesion (grey lines). The red lines show the binary rotation (1) and adhesion (0) events

**Fig. 4:**
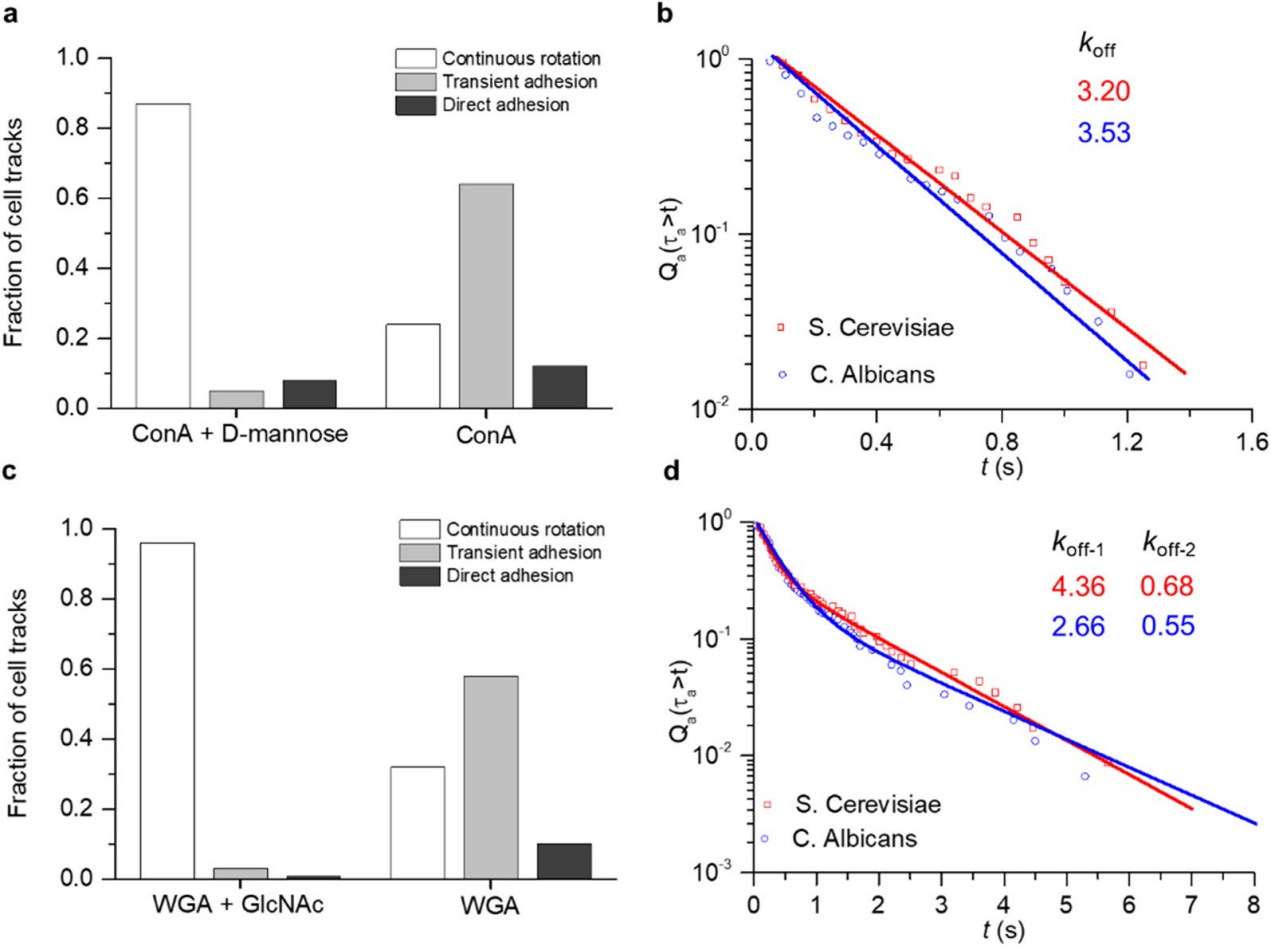
Investigation of adhesion strength of single yeast cells using scRAFA. **a** Fraction of tracked cells with a total of 40 cells that show continuous rotation, transient adhesion and direct adhesion over a tracking period of 60 s. ConA + D-mannose: The substrate is functionalized with ConA and excess D-mannose is added into the solution. ConA: The substrate is functionalized with ConA and no D-mannose is added into the solution. **b** Fraction of transient ConA-mannoside adhesion events with lifetime $\tau$_a_ ≥ *t* was plotted versus *t* for two strains of yeast cells, i.e., *S.* *Cerevisiae* and *C.* *Albicans*. *Q*_*a*_ *(*$\tau$_*a*_ ≥ *t)*: fraction of transient adhesion events with lifetime $\tau$_a_ ≥ *t*. *t* ranges from 0 to the maximum lifetime **c** Fraction of tracked cells with a total of 40 cells that show continuous rotation, transient adhesion, and direct adhesion over a tracking period of 60 s. WGA + GlcNAc: The substrate is functionalized with WGA and excess GlcNAc is added into the solution. WGA: The substrate is functionalized with WGA and no GlcNAc is added into the solution. **d** Fraction of transient WGA-chitin adhesion events with lifetime $\tau$_a_ ≥ *t* was plotted versus *t* for two strains of yeast cells, i.e., *S.* *Cerevisiae* and *C.* *Albicans*. *Q*_*a*_ *(*$\tau$_*a*_ ≥ *t)*: fraction of transient adhesion events with lifetime $\tau$_a_ ≥ *t*. *t* ranges from 0 to the maximum lifetime

In biology, cell-adhesion molecules vary and show significant difference in their effects on the duration of the cell adhesion and cell–cell interactions. To demonstrate how scRAFA quantifies the cell adhesion, we functionalize the substrate with ligands and investigate the cell-receptor-ligand interactions through tracking the light-driven cell rotation and the associated cell-substrate adhesion events. We first study the interaction between mannosides on the yeast cells and concanavalin A (ConA) immobilized on the substrate, where the mannosides are uniformly distributed on the cell surface (Additional file 1: Fig. S5). Such homogeneous yeast cells are chosen to preclude the anisotropic cell shape effect in our assay [31, 32]. Once a targeted cell was trapped and driven into rotation mode with the working laser beams, we observed three behaviors: direct adhesion (Additional file 3: Movie S2), transient adhesion (Additional file 4: Movie S3), and continuous rotation with no adhesion (Additional file 5: Movie S4). To quantify the behaviors, we retrieved the time-dependent light intensity signals from the cell images using the in situ recorded video once the cell was trapped at the laser center (Additional file 6: Movie S5). For a cell with continuous rotation, there is no adhesion between the substrate surface and cell receptors. The recorded optical oscillatory signals correspond to a continuous rotation of the cell without adhesion from 0 to 40 s (Fig. 3a). For the cell with transient adhesion, our recorded multiple transient adhesion events indicate the adhesion is mediated by a series of weak and low-affinity adhesion. The alternating oscillatory and constant signals correspond to rotation and transient adhesion events, respectively (Fig. 3b). Specifically, once the cell is trapped close to the substrate, the cell will experience continuous rotation in the initial time series (0–8 s) followed by multiple transient adhesion events (8–33 s). The cell will finally stop rotation and adhere to the substrate (33–40 s). This time-dependent behavior is like in vivo cell rolling adhesion process where the cell sliding promotes the formation of new interactions, thereby slowing dissociation and prolonging bond lifetime to reach the permanent adhesion. For the cell that directly adheres to the substrate without any rotation, there is a constant signal once the cell is trapped at the laser position (Fig. 3c). At 3.2 s, we remove the laser beam and the cell remains at the original position confirming the stable cell-substrate adhesion. For the scRAFA applications, we are mostly interested in the transient adhesion behavior, which dominates in vivo cell adhesion and cell–cell interactions. Therefore, we optimized the concentration of the ConA on the substrate to maximize the transient adhesion events (Additional file 1: Fig. S6). To prove that our observed rotation-adhesion events arise from the specific interaction between mannoside on the cell and ConA molecules on the substrate rather than other effects (e.g., non-specific interaction), we performed a control experiment in which excess D-mannose was added into the cell solution to block the mannoside on the cell membrane and thus the mannoiside-ConA interaction. As a result, most of the yeast cells in the control experiment (ConA + D-mannose), once optically trapped, underwent continuous rotation without adhesion onto the substrate (Fig. 4a).

To quantify the cell adhesion using our scRAFA, we collected the time-dependent intensity signals (Fig. 3b) for the duration that feature cell rotation with transient adhesion for 40 individual cells and analyzed the dissociation constants (*k*_off_) as a parameter of the adhesion kinetics for both non-pathogenic (*S.* *Cerevisiae*) and pathogenic (*C.* *Albicans*) yeast cells (Fig. 4b). Both strains show single exponential distribution with similar dissociate constants (*k*_*off*_), indicating both have the similar levels of manosides [31]. With the larger shear force arising from the increased laser power, the measured dissociate constant shows a higher value, revealing that the mannoiside-ConA interaction is through slip-bonds whose lifetime decreases with the increased shear force (Additional file 1: Fig. S7). We further compare our result with previously measured value by AFM [33]. Interestingly, our measured dissociation constants of mannosides for both strains are one order of magnitude larger than those in the literature [33]. We believe that this variation arises because the dissociation constants measured using scRAFA reflect the cell interaction in the lateral direction (Fig. 1d) while AFM-based measurement was carried out in the normal direction (Fig. 1b). In the presence of the flow-induced shear stress, which mimics in vivo biological process, the interacting molecules (i.e., receptors on cells and ligands on substrates) can reorientate and slip apart, becoming shorter-lived bonds and corresponding to the larger dissociation constants.

We further apply our scRAFA to successfully quantify the heterogeneous adhesion between chitin on single yeast cells and wheat germ agglutinin (WGA) immobilized on the substrate. In contrast to the uniformly distributed mannoside on the cell surface, chitins are non-uniformly distributed on the cell surface and high-concentrated chitins are localized in the bud scars (Additional file 1: Fig. S8). We first conducted a control experiment to confirm the role of specific interaction between chitin on the cell and WGA molecules on the substrate in the cell adhesion. As shown in the control experiment (WGA + GlcNAc), where excess GlcNAc was added into the cell solution to block the chitin on the cell membrane and thus the chitin-WGA interaction, most of the yeast cells underwent continuous rotation without adhesion (Fig. 4c). Then, we collected the adhesion time during the transient adhesion using the scRAFA and analyzed the survival curves of chitin for both *S.* *Cerevisiae* and *C.* *Albicans* yeast cells (Fig. 4d). The double exponential survival curves indicate that there are two types of chitin distributions along a single cell surface, which is consistent with the previous report on the heterogeneous distribution of chitins on the cell membrane [34]. *k*_*off-1,*_ corresponding to the lower adhesion of cell walls with the substrate, is approximately 5–6 times larger than *k*_*off-2,*_ corresponding to the higher adhesion of chitinous bud scars with the substrate*,* which is also consistent with previously reported data [34]. We further analyze the fitting parameters A and B of ${Q}_{a} \left({\tau }_{a}\ge \hspace{0.17em}t\right)=A{e}^{{k}_{off-1}t}+B{e}^{{k}_{off-2}t}$ (see Materials and Methods) to obtain the percentage of normal cell walls and chitinous bud scars over the whole cell area, respectively. The calculated A and B are 0.78 and 0.22, respectively, indicating the normal cell walls account for 78% of the total cell area while chitinous bud scars account for 22%. The higher adhesion associated with chitinous bud scars is also responsible for the fact that the longer transient adhesion of over 0.5 s occurs at the specific orientations of the rotating cells where the scars are closest to the substrate (Additional file 7: Movie S6).

**Fig. 5:**
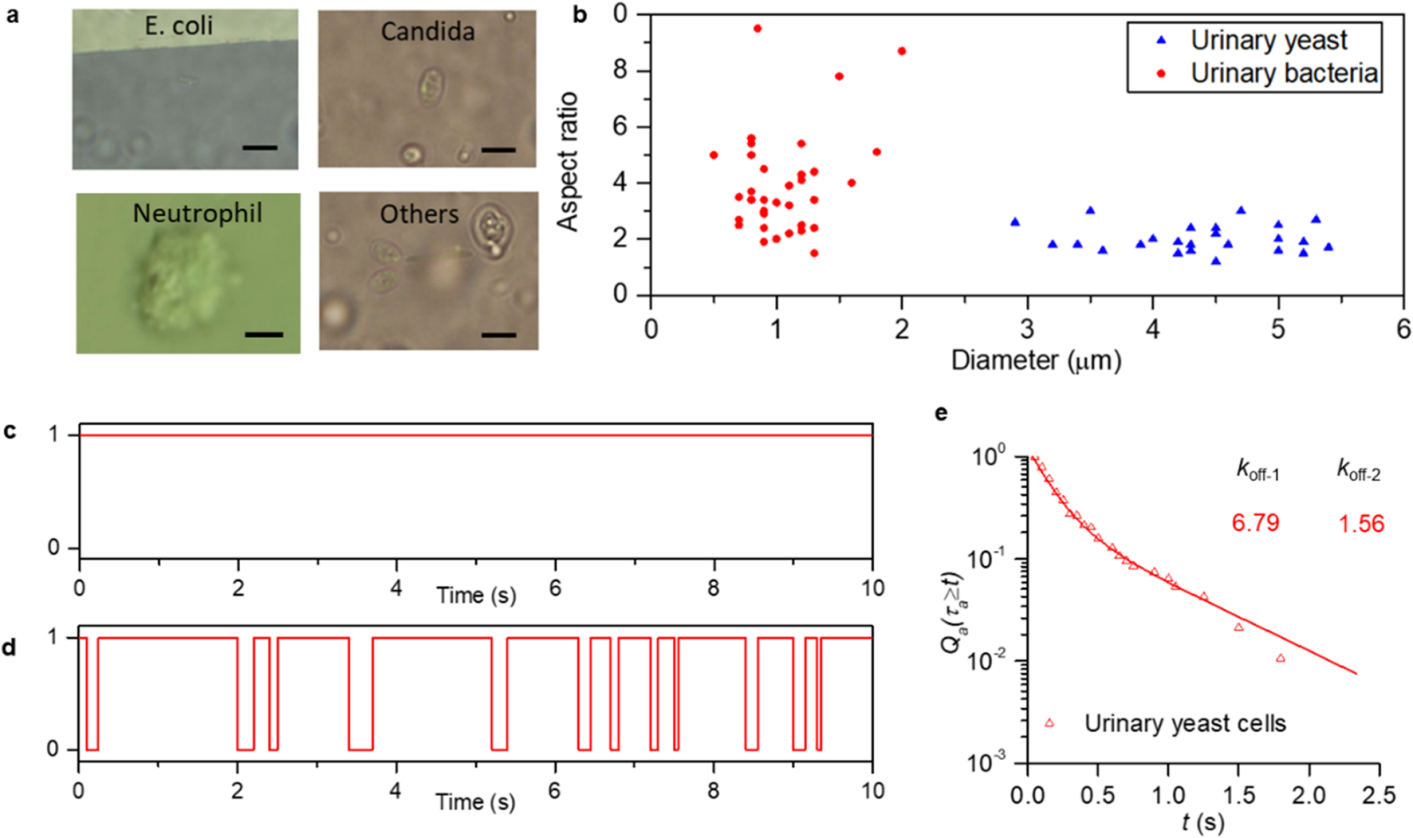
Investigation of adhesion strength of diverse cells in human samples using scRAFA. **a** Gallery of optical images of different bacteria and cells trapped and rotated in human samples during the scRAFA-based adhesion measurement. Others: Other types of urinary organisms for which we can hardly define their categories. The scale bars: 5 μm. **b** Diameter and aspect ratio distributions of bacteria and cells whose rotation has been achieved successfully by our scRAFA. **c**, **d** Two scenarios of binary rotation (1) and adhesion (0) events for fungi in human urine over a measurement period of 10 s. **e** Fraction of chitin’s transient adhesion events with lifetime $\tau$_*a*_ ≥ *t* was plotted versus *t* for urinary yeast cells. *Q*_*a*_ *(*$\tau$_*a*_ ≥ *t)*: fraction of transient adhesion events with lifetime $\tau$_*a*_ ≥ *t*. *t* ranges from 0 to the maximum lifetime. The experimental data are fitted with double exponential decay curves to extract the dissociate constants: *k*_*off-1*_ = 6.79 s^−1^ and *k*_*off-2*_ = 1.56 s^−1^ (See Materials and Methods)

Furthermore, we extend the applicability of scRAFA to measure *in-vivo*-like shear force occurring in blood vessels and urinary bladders. For this purpose, we collected a series of clinical biofluids (i.e., blood and urine) with a wide range of organisms from bacteria to neutrophils of different sizes and aspect ratios. We succeeded in trapping and rotating these organisms as required for the scRAFA-based adhesion measurement (Fig. 5a and b, Additional file 8: Movie S7). When applying scRAFA to measure the transient adhesion time of organisms in clinical samples, we noticed that multiple factors can contribute to their adhesion and thus cause inaccurate measurement of the adhesion kinetics. Specifically, many bacteria and neutrophils in the clinical samples have irregular shapes. As a result, the organism-substrate distance may vary at different organism orientations during their rotation [35, 36]. In addition, some of the bacteria and neutrophils show complex time-dependent dynamics. For example, the flagella of the bacteria can constantly change the bacterial orientation [37]. The receptors on neutrophils can also dynamically redistribute their spatial distribution on the cell membranes [38, 39]. Therefore, to acquire the more accurate force analysis and adhesion measurement, a more complex modeling of the organisms and their interactions with the substrate will be required to preclude these factors, which is beyond the scope of this report.

Despite current challenge in quantifying the adhesion forces on bacteria and neutrophils, we have successfully measured the adhesion strength of yeast cells in as-collected human urine without any pre-treatment, which is highly relevant to study of the *Candida* urinary tract infections (UTI) [40]. Our scRAFA has revealed that the urinary yeast cells exhibit diverse durations of transient adhesion (Additional file 1: Fig. S9). Some urinary yeast cells show continuous rotation (Fig. 5c), while others exhibit multiple transient adhesion events during the rotation (Fig. 5d). Optical images of these cells also indicate their different properties (Additional file 1: Fig. S10): the cell in Fig. 5c looks brighter and smoother than that in Fig. 5d. Our measured dissociate constants of mannosides and chitins receptors on urinary yeast cells (Fig. 5e, Additional file 1: Fig. S11) are larger than those of mannosides and chitins receptors on the previously measured two strains (Fig. 4), indicating that in vivo environmental conditions can significantly change the cell adhesion strength compared with the cultured environment. A possible reason for the yeast cells in human urine to have the lower mannosides and chitins adhesion strengths is the inhibition of mannosides and chitins due to their binding with urinary proteins (Additional file 1: Note S2). A comprehensive analysis of the clinical urine solutions at the different *Candida* UTI stages will be required to further prove the protein-binding effect on the cell adhesion.

## Discussion

We have developed light-driven scRAFA to measure the adhesion of individual cells in a label-free and non-contact fashion. With a range of capabilities (i.e., optical trapping, rotation, imaging, and spectroscopy) integrated in a single system, our scRAFA can pinpoint almost any targeted organism in complex fluids for the adhesion measurement along with the additional optical characterizations of the organisms’ structures and other functions, which facilitates the establishment of full structure–function relations at the single-organism level. Different from many of the existing adhesion measurements that are limited to tensile forces, our scRAFA enables rotational adhesion and shear force measurements for cells with both homogeneous and heterogeneous surfaces. The capability of revealing the structural and functional heterogeneity on single cells is crucial to studying cellular microdomain, clustering, and tether [41]. Our adhesion analysis is also instrumental in understanding immune response [42] and bacterial infection [43]. Even more impressively, our scRAFA is applicable to a wide range of organisms under the different physiological conditions. It can even reveal the shear-force-dependent adhesion behanviors due to the tunable fluidic flow and rotation torque by the laser power. Our initial study shows that the scRAFA can distinguish the adhesion behaviors between cultured yeast cells and those in clinical samples. However, the broader applications of the scRAFA to quantify the adhesion of variable organisms in clinical samples will require more complete modeling of the organisms and their interactions with substrates. With its superior performance and general applicability, scRAFA, once fully developed, is expected to play a critical role in a wide range of fields from cell biology to immunotherapy to urinary tract infection.

## Materials and methods

### Optical setup for scRAFA

Our experimental setup for scRAFA is shown in Fig. 2a. It consists of an inverted optical microscope (Nikon) coupled with a 532 nm laser (Coherent, Genesis MX 71 STM-1 W) and a 785 nm laser (Sacher Lasertechnik, TEC 510). The intensities and polarizations of both laser beams are tuned by optical density filters (Thorlabs, NE10A) and half-wave plates (Thorlabs, WPHSM05-532 and WPHSM05-780), respectively. The beam diameters are adjusted by beam expanders (Thorlabs, GBE05-A for 532 nm, GBE05-B for 785 nm) and focused on the substrate using an oil objective (× 100, NA = 1.3) in the microscope. The beam waists at the focal planes are ~ 600-1000 nm. Sample movement is controlled by an automatic translation stage (Prior, Proscan III). Optical images of the rotating cells are acquired with a CMOS camera (Nikon, DS-Fi3). The camera operates at 20 frames per second with 720 × 512 pixels when collecting data for cell adhesion analysis. Two notch filters are placed on the beam splitter cages to block the back reflection of 785 nm and 532 nm laser beams.

### Fabrication of light-absorbing substrates

Substrates used for scRAFA, which strongly absorb the 532 nm laser beam while being mostly transparent to the 785 nm laser beam, are fabricated in multiple steps. Firstly, glass coverslips (Cadinal Health, M6047-10A) were sonicated in acetone and isopropyl alcohol solution for 5 min. Secondly, the coverslips were rinsed with deionized water and blown dry with nitrogen gas. Thirdly, the coverslips were mounted into the thermal evaporator (Cooke Evaporator) and 4.5 nm Au films were thermally deposited on the coverslips at a base pressure of 1 × 10^–5^ torr and a deposition speed of 2 Å/s. Finally, after the Au deposition, the samples were thermally annealed at 550 °C for 2 h in a thermal chamber (Thermal Fisher, Lindberg/Blue M) to work as the substrates.

### Preparation of biological samples

*S.* *cerevisiae* (S288C) and *C.* *albicans* (SC5314) were cultured in YPD agar solution (2% agar, 2% peptone, and 1% yeast extract) at 37 °C. Before their uses in the experiments, all the cells were washed three times with phosphate-buffered saline and resuspended in YPD to have a final concentration of 10^5^ cells/mL. To block the chitins molecules on the cell surfaces, 200 mM of *N*-acetylglucosamine was added into the cell solution.

Collected human urine was injected into falcon tubes. After 10 min, most organisms (i.e., bacteria and cells) were settled down to the tube bottom. We took the upper volume of the urine solution and injected it into a microfluidic chip for analysis. Clinical human blood sample was purchased in Zenbio Inc. Neutrophiles were collected directly from the blood sample through histopaque double density centrifugation. Briefly, 3 mL histopaque 1119, 3 mL histopaque 1077, and 6 mL whole blood solution were added sequentially to a 15 mL falcon tube, followed by centrifugation at 400 × g for 30 min. By removing the plasma and histopaque layers, the neutrophiles were collected and injected into the microfluidic chip for analysis.

### scRAFA measurement procedure

All the substrates were washed with deionized water and dried with nitrogen, followed by treatment with oxygen plasma for 5 min. The substrates were incubated with 500 μg/mL WGA or 100 μg/mL ConA in a tissue culture petri dish for 60 min at 37 °C. Next, the substrates were washed with phosphate-buffered saline to remove the unbound proteins. A silicone adhesive spacer with 0.12 mm in depth was attached to the functionalized substrates. We punched two holes on the spacer as inlets and outlets of the microfluidic channel with a length of 13 mm. Another cleaned glass coverslip was used to seal the microfluidic chamber. The sample was then placed on the stage of an inverted optical microscope and connected to a syringe pump. The cell solution (~ 20 μL) was injected into a microfluidic chip through a syringe with a flow rate of 0.1 μL/s. After the chamber was fully filled with a liquid solution, we waited for 10 s to let the solution stabilize and then turned on the lasers to conduct the scRAFA experiments.

After collecting the lifetimes of transient adhesion for 40 cells, we analyzed the data by Kaplan–Meier method (a built-in algorithm in Matlab) to calculate the fraction of transient adhesion lasting for at least the targeted duration, which are shown as scatter plots in Figs. 4b, d. An exponential probabilistic model was used to fit the scatter plots. The maximum likelihood estimation (a built-in algorithm in Matlab) was used to calculate the model parameters. Specifically, the adhesion of chitins was fitted with double exponential curves:

1 $${Q}_{a} \left({\tau }_{a}\ge \hspace{0.17em}t\right)=A{e}^{{k}_{off-1}t}+B{e}^{{k}_{off-2}t}$$

where A and B are the fitting parameters, while *k*_*off-1*_ and *k*_*off-2*_ are the dissociate constants.

The adhesion of mannosides was fitted with a single exponential curve:

2 $${Q}_{a} \left({\tau }_{a}\ge \hspace{0.17em}t\right)={e}^{{k}_{off}t}$$

where *k*_*off*_ is the dissociate constant.

### Fluorescence labeling and imaging

Yeast cells were washed three times by phosphate-buffered saline, followed by an injection of 1 mg/mL FITC-labeled WGA and ConA. After incubation for 40 min, the yeast cells were centrifugated at 3000 revolutions per minute for 3 min. After removing the solution containing the extra fluorescence molecules, we resuspended the cells in the phosphate-buffered saline solution. Microscope well slides were washed three times and mounted on the microscope. The cells were injected into the microscope well slides for fluorescence imaging.

### Multiparticle collision dynamics simulation

To bridge the dimension gap between the microscopic cells and the fluid molecules, we have employed a hybrid simulation scheme. The fluid was described by multiparticle collision dynamics (MPC), while a cell and its interaction with the fluid were simulated by molecular dynamics. In MPC, the fluid was coarse-grained into a large number of point-like particles with a simple collision rule by which mass, linear momentum, angular momentum and energy can be locally conserved. The MPC algorithm can properly capture hydrodynamic interactions, thermal fluctuations, mass transport and heat conduction, and has been widely used in studies of complex fluids. Standard MPC parameters were employed in simulations, which produced liquid-like dynamics. For simplicity, the cell was modelled as a rigid bead and coupled with the fluid particles through the Lennard–Jones-type potential and non-slip boundary condition, which naturally led to positive thermophoresis (i.e., thermophobic) under a temperature gradient.

Moreover, we used a local thermostat to create a high-temperature area, mimicking the optical heating, and used a mesoscale phoretic osmotic boundary to generate the thermo-osmotic flow along the substrate.

### Temperature simulation and measurement

We simulated temperature distribution at a laser-irradiated substrate using the finite element method (COMSOL Multiphysics). A 2D axis-symmetric model comprising a substrate and water surrounding was established. The model utilized pre-loaded modules of heat transfer in solids, liquids, and non-isothermal laminar flow coupled with conjugate heat transfer physics. Laser power absorbed by the substrate was modelled as Gaussian heat influx and was coupled to heat transfer in water using temperature continuity at the water/substrate interface surface. The thermal conductivity of a thermally annealed 4.5 nm gold film on a glass coverslip is low. The absorption coefficient (30%) of the annealed gold film was incorporated into the simulation whose value was the same as experimentally measured. Other boundaries were maintained at room temperature of 293 K.

In situ temperature measurement at a laser-irradiated substrate was conducted through commercial thermal imaging with quadriwave shearing interferometry. Briefly, a thermal imaging camera (SID4-HR, Phasics) was mounted to an inverted optical microscope (Nikon Ti-E) with an × 100 oil objective. A computer-controlled LabVIEW software (Sidfthermo, Phasics) was used to collect optical phase images and convert the images to thermal profiles based on the temperature-dependent refractive index of the substrate.

## Supplementary Information

**Additional file 1: Figure S1.** *X-y* plane (see Fig. 2) temperature profiles at the focal point of a heating laser beam directed onto a light-absorbing substrate. (a) Measured temperature profile. (b) Simulated temperature profile. The intensity of a 532 nm laser beam is 0.2 mW/μm^2^ and the beam diameter is 0.8 μm. Scale bars: 3 μm. **Figure S2.** Force analysis of an optothermally trapped cell with two laser beams. (a) Schematic illustration of a trapped cell with all the relevant forces. The trapped cell is balanced by thermoosmotic (F_TO_), thermophoretic (F_TP_) and optical forces (F_o_). (b) Simulated cell distribution under the balance of thermoosmotic (F_TO_), thermophoretic (F_TP_) and optical forces (F_o_). The zero position refers to the laser beam center. k_o_ is the spring constant of the optical force. With the higher optical force, the cell moves closer to the laser beam center. Without an optical force (i.e., k_o_ = 0), the cell cannot be stably trapped (see black line). **Figure S3.** Trajectory of an optothermally trapped cell. (a) A temporal trajectory in *x-y* plane of the center of a rotating cell relative to the laser beam center (0, 0). The optical imaging duration is 60 s. (b) Histogram with Gaussian fitting of the radial distance from the cell center to the laser beam center. **Figure S4.** Simulated *x-y* plane temperature profiles at the focal point of a heating laser beam directed onto a light-absorbing substrate using 532 nm laser. The intensity of a 532 nm laser beam is 0.2 mW/μm^2^. Scale bars: 1 μm. The star symbol represents the highest temperature point on the cells, which is below 30 degrees. **Figure S5.** Fluorescence images of FITC-ConA-labelled yeast cells, which show the uniform distribution of the mannosides. The scale bars are 3 μm. **Figure S6.** Fraction of tracked cells that show continuous rotation, transient adhesion, and direct adhesion over a tracking period of 60 s. The substrates were incubated with 10, 50, 100, 200 μg/mL ConA in tissue culture petri dish for 60 min at 37 °С, respectively. **Figure S7.** Measured dissociation affinity (k_off_) of the trapped cell (*S. Cerevisiae*) under different optical powers of 532 nm laser. The optical power of 785 nm laser is 1 mW/μm^2^ in all studies. **Figure S8.** Fluorescence images of two strains of yeast cells labelled with FITC-WGA: (a) *S. Cerevisiae* and (b) *C. Albicans*. Scale bars: 3 μm. **Figure S9.** Distributions of the duration of transient adhesion for selected 20 individual yeast cells in human urine over a measurement period of 15 s. (a) Duration of transient adhesion events for mannosides receptors. (b) Duration of transient adhesion events for chitin receptors. The 15 s was the longest period during which no other organisms entered the trapping center to disrupt the single-cell analysis by scRAFA. **Figure S10.** Optical images of rotating yeast cells in human urine. (a) Optical image of a rotating cell in Fig. 5c. (b) Optical image of a rotating cell in Fig. 5d. Scale bars: 3 μm. **Figure S11.** Measurement of adhesion strength of mannosides for urinary yeast cells by scRAFA. Fraction of transient adhesion events with lifetime ($\tau$_a_) ≥ *t* was plotted versus t for urinary yeast cells. Q_a_ ($\tau$_a_≥ *t*): fraction of transient adhesion events with lifetime ($\tau$_a_) ≥ *t*. *t*: adhesion lifetime. The data are fitted with single exponential decay curves to extract the dissociate constant. The dissociate constant (k_off_) is 6.32 s^−1^. **Note S1.** Determining the cell-substrate interaction distance. **Note S2.** Explaining why yeast cells in human urine exhibit the lower adhesion than the yeast cells cultured in laboratory.

**Additional file 2: Movie S1.** Single cell trapping and rotation.

**Additional file 3: Movie S2.** Single cell direct adhesion.

**Additional file 4: Movie S3.** Single cell transient adhesion.

**Additional file 5: Movie S4.** Single cell continuous rotation.

**Additional file 6: Movie S5.** Tracking single cell rotation and adhesion.

**Additional file 7: Movie S6.** The adhesion happens at a specific orientation.

**Additional file 8: Movie S7.** General applicability of scRAFA in clinical solutions.

## Data Availability

All data generated or analysed during this study are included in this published article and its Additional files.
