# Supplementary material for: Light-driven single-cell rotational adhesion frequency assay
Source: eLight. 2022 Aug 8;2(1):13. doi: 10.1186/s43593-022-00020-4 (PMC9358104; doi:10.1186/s43593-022-00020-4)
Supplement: Supplementary file 1 — Additional file 1: Figure S1. X-y plane (see Fig. 2) temperature profiles at the focal point of a heating laser beam directed onto a light-absorbing substrate. (a) Measured temperature profile. (b) Simulated temperature profile. The intensity of a 532 nm laser beam is 0.2 mW/μm2 and the beam diameter is 0.8 μm. Scale bars: 3 μm. Figure S2. Force analysis of an optothermally trapped cell with two laser beams. (a) Schematic illustration of a trapped cell with all the relevant forces. The trapped cell is balanced by thermoosmotic (FTO), thermophoretic (FTP) and optical forces (Fo). (b) Simulated cell distribution under the balance of thermoosmotic (FTO), thermophoretic (FTP) and optical forces (Fo). The zero position refers to the laser beam center. ko is the spring constant of the optical force. With the higher optical force, the cell moves closer to the laser beam center. Without an optical force (i.e., ko = 0), the cell cannot be stably trapped (see black line). Figure S3. Trajectory of an optothermally trapped cell. (a) A temporal trajectory in x-y plane of the center of a rotating cell relative to the laser beam center (0, 0). The optical imaging duration is 60 s. (b) Histogram with Gaussian fitting of the radial distance from the cell center to the laser beam center. Figure S4. Simulated x-y plane temperature profiles at the focal point of a heating laser beam directed onto a light-absorbing substrate using 532 nm laser. The intensity of a 532 nm laser beam is 0.2 mW/μm2. Scale bars: 1 μm. The star symbol represents the highest temperature point on the cells, which is below 30 degrees. Figure S5. Fluorescence images of FITC-ConA-labelled yeast cells, which show the uniform distribution of the mannosides. The scale bars are 3 μm. Figure S6. Fraction of tracked cells that show continuous rotation, transient adhesion, and direct adhesion over a tracking period of 60 s. The substrates were incubated with 10, 50, 100, 200 μg/mL ConA in tissue culture petri dish for 60 [file 43593_2022_20_MOESM1_ESM.docx]

**Supplementary Information**

Light-driven single-cell rotational adhesion frequency assay

Yaoran Liu^1^, Hongru Ding^2^, Jingang Li^3^, Xin Lou^4^, Mingcheng Yang^4,5,6^, and Yuebing Zheng^1,2,3,7*^

**Affiliations:**

^1^Department of Electrical and Computer Engineering, The University of Texas at Austin, Austin, TX 78712, USA

^2^Walker Department of Mechanical Engineering, The University of Texas at Austin, Austin, TX 78712, USA

^3^Materials Science & Engineering Program and Texas Materials Institute, The University of Texas at Austin, Austin, TX 78712, USA

^4^School of Physical Sciences, University of Chinese Academy of Sciences, Beijing, 100049, China

^5^Beijing National Laboratory for Condensed Matter Physics and Laboratory of Soft Matter Physics, Institute of Physics, Chinese Academy of Sciences, Beijing, 100190, China

^6^Songshan Lake Materials Laboratory, Dongguan, Guangdong, 523808, China

^7^Department of Biomedical Engineering, The University of Texas at Austin, Austin, TX 78712, USA

*Corresponding author: Yuebing Zheng.

**Email:** [zheng@austin.utexas.edu](mailto:zheng@austin.utexas.edu)

Supplementary Figures and Notes


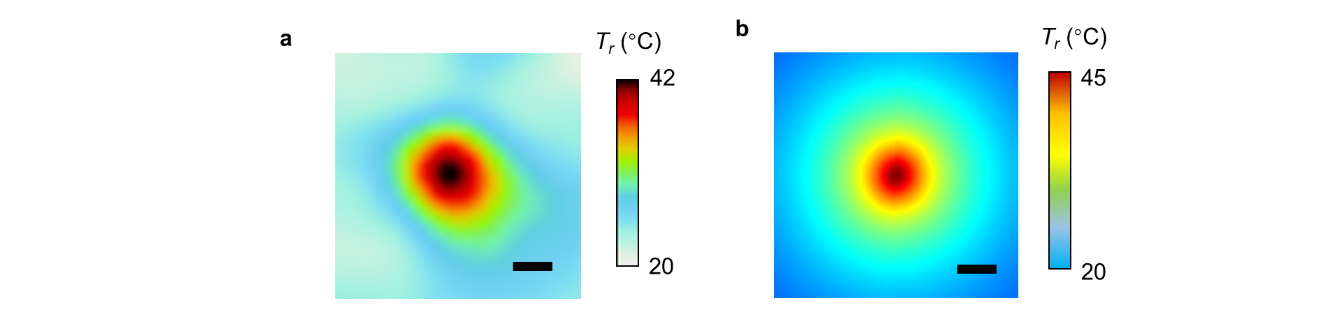


**Fig. S1.**

***X-y* plane (see Fig. 2) temperature profiles at the focal point of a heating laser beam directed onto a light-absorbing substrate. (a)** Measured temperature profile. **(b)** Simulated temperature profile. The intensity of a 532 nm laser beam is 0.2 mW/μm^2^ and the beam diameter is 0.8 μm. Scale bars: 3 μm.


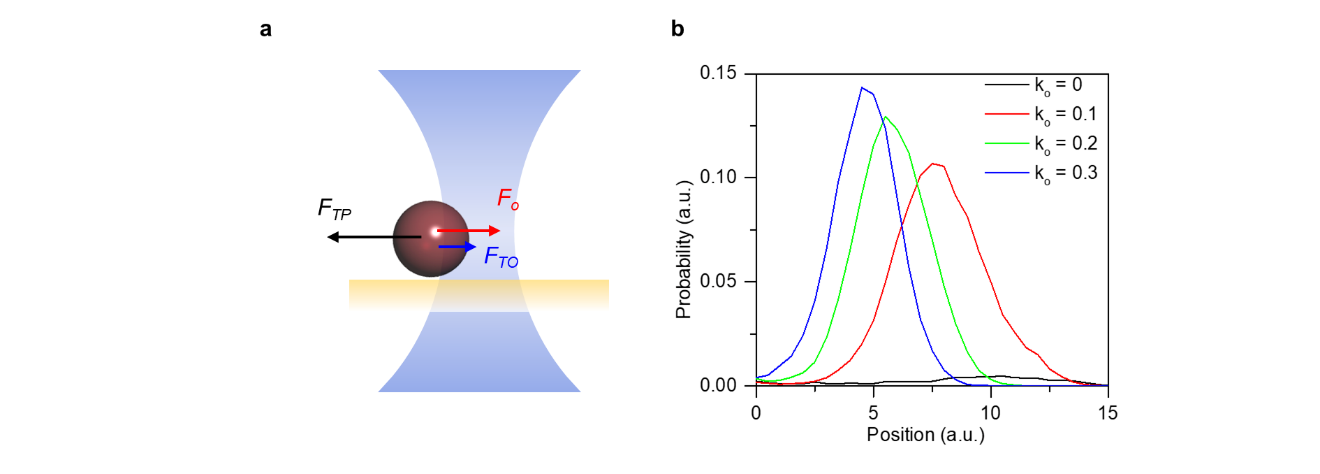


**Fig. S2.**

**Force analysis of an optothermally trapped cell with two laser beams. (a)** Schematic illustration of a trapped cell with all the relevant forces. The trapped cell is balanced by thermoosmotic (*F_TO_*), thermophoretic (*F_TP_*) and optical forces (*F_o_*). **(b)** Simulated cell distribution under the balance of thermoosmotic (*F_TO_*), thermophoretic (*F_TP_*) and optical forces (*F_o_*). The zero position refers to the laser beam center. *k_o_* is the spring constant of the optical force. With the higher optical force, the cell moves closer to the laser beam center. Without an optical force (i.e., *k_o_* = 0), the cell cannot be stably trapped (see black line).


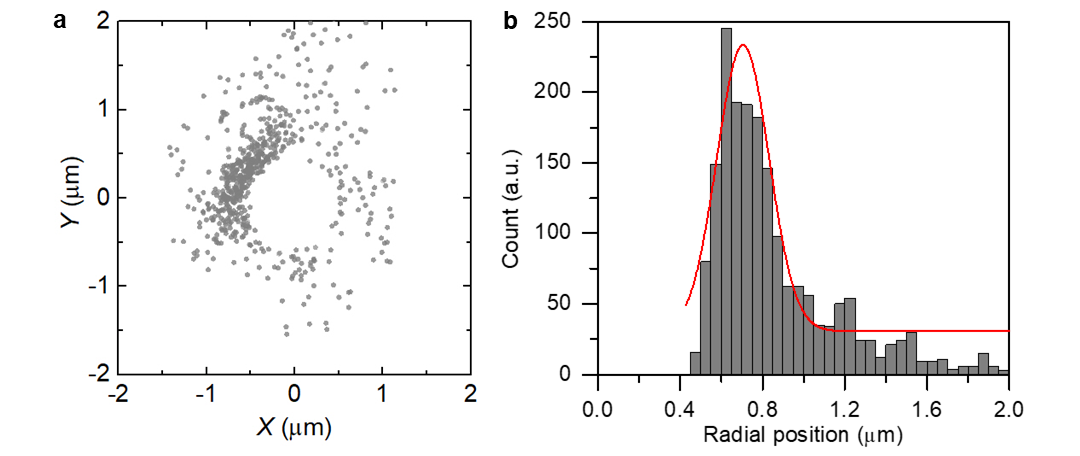


**Fig. S3.**

**Trajectory of an optothermally trapped cell.** **(a)** A temporal trajectory in *x-y* plane of the center of a rotating cell relative to the laser beam center (0, 0). The optical imaging duration is 60 s. **(b)** Histogram with Gaussian fitting of the radial distance from the cell center to the laser beam center.


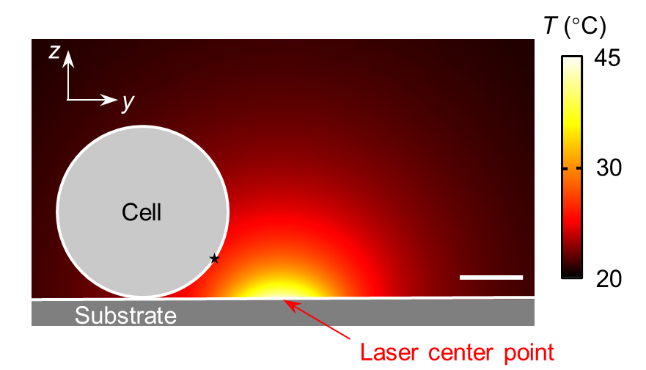


**Fig. S4.**

**Simulated *x-y* plane temperature profiles at the focal point of a heating laser beam directed onto a light-absorbing substrate using 532 nm laser.** The intensity of a 532 nm laser beam is 0.2 mW/μm^2^. Scale bars: 1 μm. The star symbol represents the highest temperature point on the cells, which is below 30 degrees.


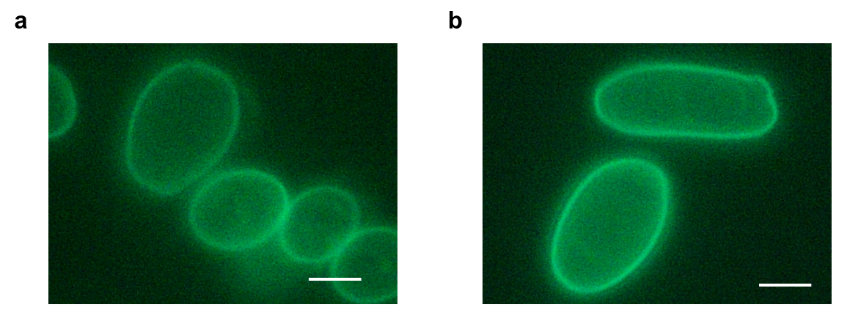


**Fig. S5.**

**Fluorescence images of FITC-ConA-labelled yeast cells, which show the uniform distribution of the mannosides.** The scale bars are 3 μm.


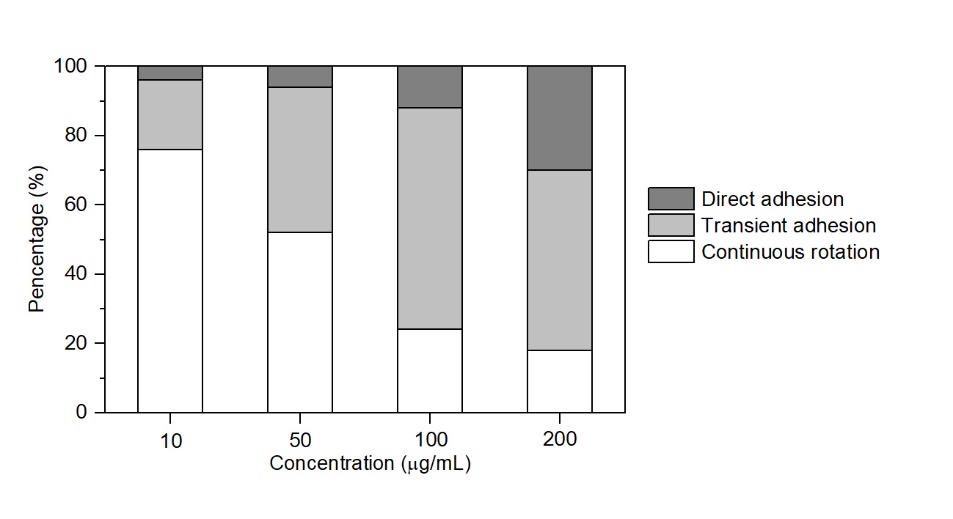


**Fig. S6.**

**Fraction of tracked cells that show continuous rotation, transient adhesion, and direct adhesion over a tracking period of 60 s.** The substrates were incubated with 10, 50, 100, 200 μg/mL ConA in tissue culture petri dish for 60 min at 37 °С, respectively.


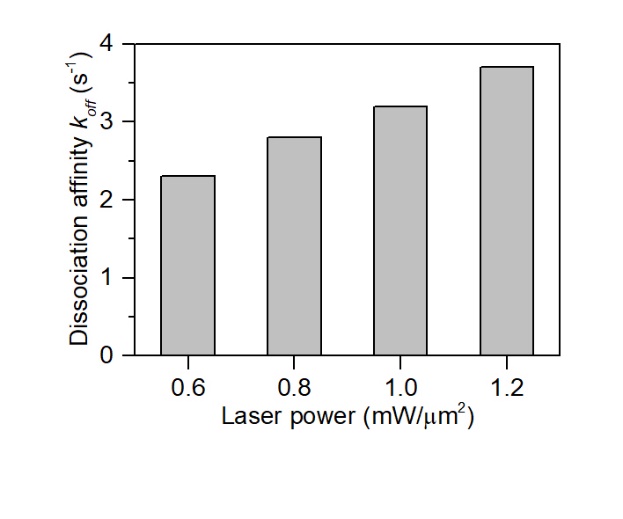


**Fig. S7.**

**Measured dissociation affinity (*k_off_*) of the trapped cell (*S. Cerevisiae*) under different optical powers of 532 nm laser.** The optical power of 785 nm laser is 1 mW/μm^2^ in all studies.


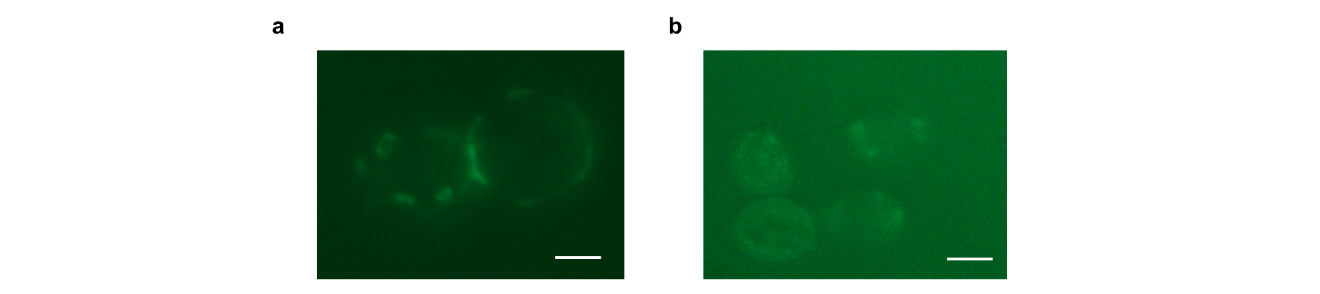


**Fig. S8.**

**Fluorescence images of two strains of yeast cells labelled with FITC-WGA: (a)** *S. Cerevisiae* and **(b)** *C. Albicans*. Scale bars: 3 μm.


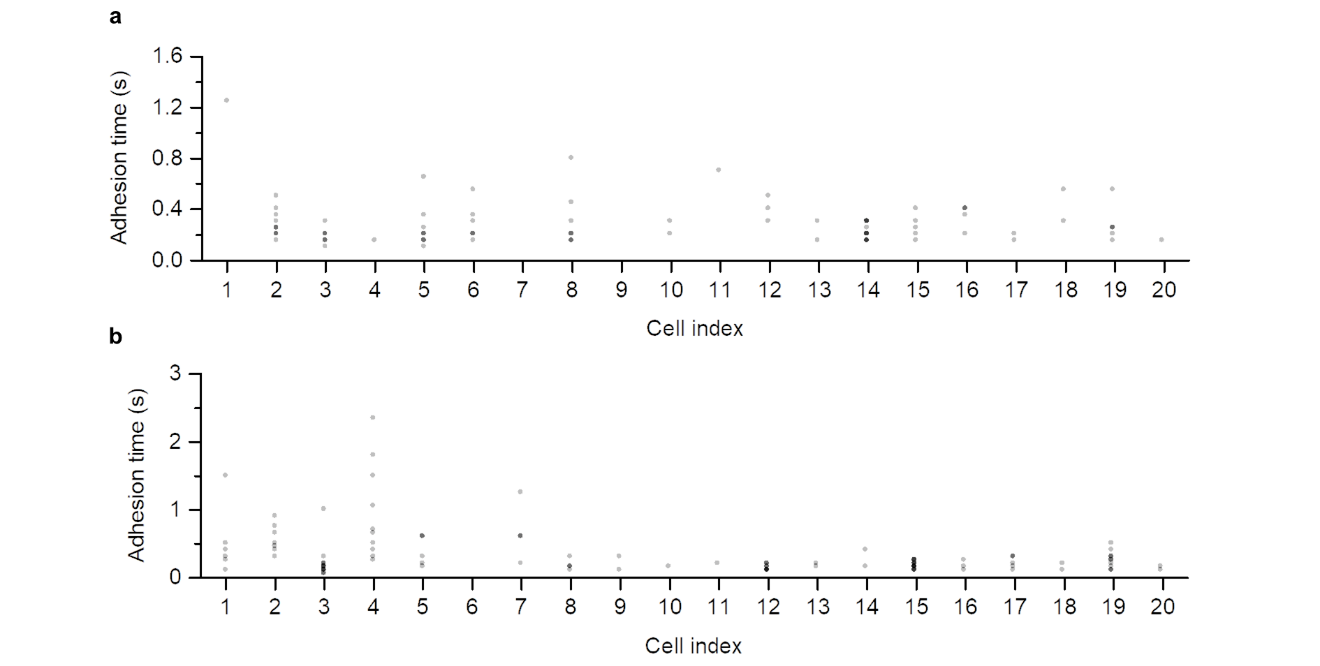


**Fig. S9.**

**Distributions of the duration of transient adhesion for selected 20 individual yeast cells in human urine over a measurement period of 15 s. (a)** Duration of transient adhesion events for mannosides receptors. **(b)** Duration of transient adhesion events for chitin receptors. The 15 s was the longest period during which no other organisms entered the trapping center to disrupt the single-cell analysis by scRAFA.


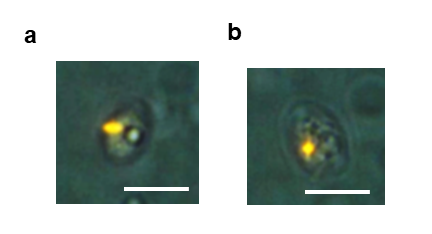


**Fig. S10.**

**Optical images of rotating yeast cells in human urine. (a)** Optical image of a rotating cell in Fig. 5c. **(b)** Optical image of a rotating cell in Fig. 5d. Scale bars: 3 μm.


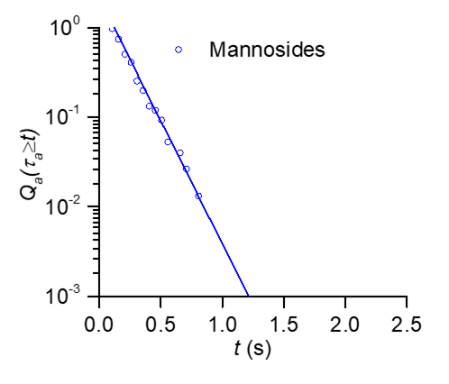


**Fig. S11.**

**Measurement of adhesion strength of mannosides for urinary yeast cells by scRAFA.** Fraction of transient adhesion events with lifetime ($\tau$ _a_) ≥ *t* was plotted versus *t* for urinary yeast cells. *Q_a_ (*$\tau$ *_a_≥ t)*: fraction of transient adhesion events with lifetime ($\tau$ _a_) ≥ *t*. *t*: adhesion lifetime. The data are fitted with single exponential decay curves to extract the dissociate constant. The dissociate constant (*k_off_*) is 6.32 s^-1^.

**Note S1: Determining the cell-substrate interaction distance**

We use plasmonic effects of the absorbing substrate to *in situ* detect the cell-substrate distance. Briefly, the localized surface plasmon resonance field of the absorbing substrate is sensitive to the surrounding’s refractive index change within the decay length of 100 nm. An increase of refractive index, which is caused by trapping of a cell close to the substrate in the current case, can induce spectral red shift (*Δλ*) of the transmission peak or dip at the resonance wavelength^1^. The closer distance between the cell and substrate, the larger the red shift observed in the transmission peak or dip. The distance between the cell and the substrate can be extracted once there is a *Δλ* match between simulated and experimental results.

We first measure the transmission spectra of the absorbing substrate without cells. Once a cell is trapped and rotated, we measure the transmission spectra again and calculate the Δλ (**Supplementary Note Fig. 1a**).

Next, we conduct electromagnetic simulation to mimic the experimental conditions. To model a cell in the solution, three-layer structure is built above the substrate (**The inset of Supplementary Note Fig. 1b**). The first layer is water layer with a refractive index of 1.33, the thickness of which corresponds to the cell distance to the substrate (*h*). The second layer is the cell membrane with a thickness of 10 nm and a refractive index of 1.46. The third layer is the cell cytoplasm with a thickness of 8 μm and a refractive index of 1.37^2^. We simulate the transmission spectra of the absorbing substrate with and without cells. As a demonstration, the transmission spectral dip with a cell shows a red shift of 8 nm when *h* is 4 nm compared to the transmission spectral dip without a cell (**Supplementary Note Fig. 1b**). By sweeping *h*, we show that the spectral shift has different values. Specifically, when *d* = 7.4 nm, the simulated spectral red shift matches well with the experimental data (**Fig. 2g**). Therefore, we estimate that the distance between the cell and substrate is 7.4 nm.


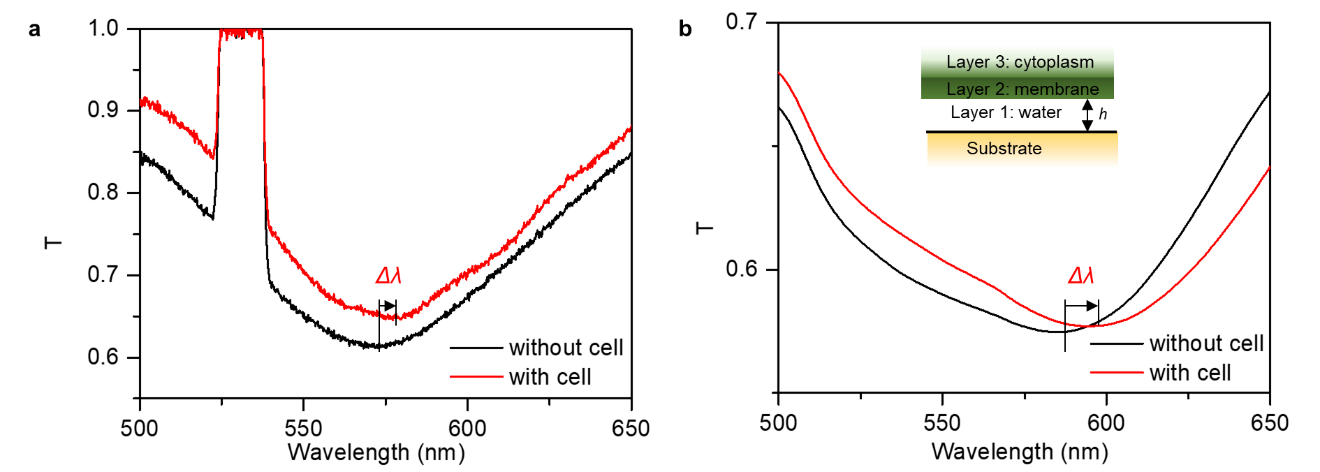


**Supplementary Note Fig. S1.**

**Interaction distance between cell and substrate. (a)** Measured transmission spectra of the absorbing substrate with and without a trapped cell. **(b)** Simulated transmission spectra of the absorbing substrate with and without a trapped cell. The distance between the cell and substrate (*h*) is 4 nm.

**Note S2: Explaining why yeast cells in human urine exhibit the lower adhesion than the yeast cells cultured in laboratory.**

Different from the yeast cells cultured in laboratory, year cells in clinical urine have the more complex liquid medium containing millions of biomolecules. Specifically, urine contains glycoproteins (90 kDa, 70 kDa, and 40 kDa) with (GlcNAc)_n_ or oligosaccharide structures^3^. Oligosaccharides are found to be routinely excreted in the urine. The concentration of the oligosaccharides varies among different people. Accumulation of oligosaccharides might be related to the glycoprotein storage disorders due to deficiencies of enzymes^4^. The urinary oligosaccharide content in galactosialidosis and sialidosis patients is found to be 1-30 nM/mg creatinine^5^. Therefore, similar to our control experiments with excess GlcNAc (Fig. 4c), the abundant oligosaccharides in the urine can bind to the yeast surfaces and reduce their adhesion to the ligand-functionalized substrates.

Meanwhile, it has been found that urine can affect growth and orientation of surface receptors on bacteria. For example, urea can induce bacterium fimbriae synthesis, which help the bacterium adhesion in urinary tract infection^6^. On the other hand, urine can also inhibit bacterium adhesion when it contains certain inhibitors, which leads to a bias in cellular receptors (i.e., fimS) orientation of bacteria^7^. Such inhibition effects of urine on surface receptors of yeast cells might lower the adhesion of yeast cells in human urine.

**Reference**

1 Hackett, L., Seo, S., Kim, S., Goddard, L. & Liu, G. Label-free cell-substrate adhesion imaging on plasmonic nanocup arrays. *Biomed. Opt. Express* **8**, 1139-1151 (2017).

2 Jung, L. S., Campbell, C. T., Chinowsky, T. M., Mar, M. N. & Yee, S. S. Quantitative interpretation of the response of surface plasmon resonance sensors to adsorbed films. *Langmuir* **14**, 5636-5648 (1998).

3 Zhu, H. *et al.* Glycopatterns of urinary protein as new potential diagnosis indicators for diabetic nephropathy. *J. Diabetes Res.* **14**, 5728087 (2017).

4 Peelen, G., De Jong, J. & Wevers, R. A. HPLC analysis of oligosaccharides in urine from oligosaccharidosis patients. *Clin. Chem.* **40**, 914-921 (1994).

5 Takahashi, Y., Nakamura, Y., Yamaguchi, S. & Orii, T. Urinary oligosaccharide excretion and severity of galactosialidosis and sialidosis. *Clin. Chim. Acta* **203**, 199-210 (1991).

6 Subashchandrabose, S. *et al.* Host-specific induction of Escherichia coli fitness genes during human urinary tract infection. *Proc. Natl. Acad. Sci.* **111**, 18327-18332 (2014).

7 Greene, S. E., Hibbing, M. E., Janetka, J., Chen, S. L. & Hultgren, S. J. Human urine decreases function and expression of type 1 pili in uropathogenic Escherichia coli. *MBio* **6**, e00820-15 (2015).
